# Working alongside next of kin to enhance discharge: A quality improvement collaboration to co-design discharge for mental health patients

**DOI:** 10.1177/10398562231198141

**Published:** 2023-08-25

**Authors:** Warren Kealy-Bateman, Daniel Stewart, Hugh Powell, Kirbie Storrier, Margo Mackenzie, Christopher Dowton

**Affiliations:** 72517 Dubbo Base Hospital, Dubbo, NSW, Australia; and; 218517 The University of Sydney School of Rural Health – Dubbo Campus, Dubbo, NSW, Australia; Graduate School of Medicine, 8691 University of Wollongong, Wollongong, NSW, Australia; 72517 Dubbo Base Hospital, Dubbo, NSW, Australia

**Keywords:** co-design, discharge, emergency department, psychiatric care, next of kin

## Abstract

**Objectives:**

Mental health (MH) patients seen in the emergency department (ED) setting are often viewed in isolation, outside of the context of their loved ones, the next of kin (NOK). This is especially problematic when a patient is detained under the mental health act. This project aimed to improve this engagement.

**Methods:**

A sense of urgency was created from a guiding coalition of the local MH and ED executive of a rural hospital. The vision was communicated to the team for action. This was then institutionally incorporated as best practice during a 3 month trial.

**Results:**

NOK were engaged more frequently as a result of this quality improvement strategy, rising to 90.8% (2021) from 63.2% (2020) compared to the previous year χ2 (1, *N*=166) =18.75, *p* = .000015. Admissions for all MH patients from the emergency department fell to 28.3% (2021) from 39% (2020) χ2 (1, *N*=652) =8.32, *p* = .0039.

**Conclusions:**

Working with NOK is a best practice strategy that was embraced by clinicians when highlighted. This resulted in less restrictive practice and more community treatment. Creating a frame for the patient that is standardised, provides containment and co-designs future health care is beneficial.

In 2021 the health district received feedback from family members. Their relatives had been seen in the emergency department (ED) and the focus was a mental health (MH) problem. Relatives felt excluded from the process of providing care for their loved one who was then discharged from the ED. There was also the suggestion, that critical subsequent events could have been avoided if they had been engaged in care. Patients who are unable, or unwilling, to identify any supports at a critical time are potentially isolated. They may also be regressed in their psychological defences, prompting concern.

In contrast, quality improvement activities had identified that 97.3% of all MH inpatients could identify a next of kin (NOK), when asked, and this person was able to meaningfully contribute to the discharge process.^1^

The patient group of most concern in the ED were those detained under the Mental Health Act NSW 2007 (MHA).^2^ In a 3 month period in 2020, 34.3% (114 of 332) of MH patients presented under the Mental Health Act Victoria 2014 to an ED in Melbourne.^3^ Not all MH patients seen in the ED for assessment are admitted; discharge home is the most common occurrence, followed by admission for MH care and less commonly a medical admission.^3^

Suicide, while rare, is a risk across multiple mental health, drug and alcohol (MHDA) diagnoses with suicidality common in the ED setting.^4^ The opportunity for psychological ‘holding’ and ‘containing’ potentially reduces this risk and may be lost if NOK are not engaged for a vulnerable patient.^5,6^ A cross sectional assessment of a patient in isolation and discharge into a void without support may utilise the prism of the MHA too rigidly, placing the patient at risk once they leave the hospital. The onus should be on the clinician to lead the process and ask the patient to identify a person who can offer that framework of safety. This is indicated under the MHA (see Table 1) as a surety but not necessarily as an absolute.

**Table 1.** Relevant provisions of the mental health act NSW 2007^2^

Section 75
Notification to carers of initial detention
(1) An authorised medical officer must, not later than 24 h after a person is detained in a mental health facility, take all reasonably practicable steps to notify any designated carer and the principal care provider (if the principal care provider is not a designated carer) of the person that the person is detained in the facility.
(2) Notice need not be given if the person is discharged or classified as a voluntary patient within that period.
Section 78
Notifications to designated carers and principal care providers of events affecting patients or detained persons
(1) An authorised medical officer of a mental health facility must take all reasonably practicable steps to notify any designated carer and the principal care provider (if the principal care provider is not a designated carer) of a patient or person detained in the facility if any of the following events occurs—
(a) The patient or person is absent from the facility without permission or fails to return at the end of a period of leave,
(b) It is proposed to transfer the patient or person, or the patient or person is transferred, to another mental health facility or other facility,
(c) The patient or person is discharged from the mental health facility,
(d) The patient or person is re-classified as a voluntary patient,
(e) It is proposed to apply to the Tribunal for an ECT inquiry under Part 2 or to ascertain whether the patient or person is capable of giving informed consent to electro convulsive therapy,
(f) A surgical operation is performed on the patient or person under Part 3,
(g) It is proposed to apply to the Secretary or the Tribunal for consent to a surgical operation or special medical treatment under Part 3,
(h) The patient or person has any matter before the Tribunal.
(2) The authorised medical officer must give the notice as soon as practicable after becoming aware that the event has occurred.
(3) In the case of a proposed transfer, the notice must be given before the relevant order or arrangement is made, except in an emergency.

The team noted the pressures on the service and the local resources. It takes more time to work with others. Yet also noted were the benefits to patients.

The specific purpose of this project was to work with ED patients who were under the MHA and engage their NOK in the process of discharge planning.

## Methods

Between July 2018 and June 2019 there were 11,254 urgent and 15,338 semi-urgent presentations to this rural hospital emergency department.^7^

In June 2021 the local MH and ED executive raised the project of Working Alongside Next of kin To Enhance Discharge (WANTED) with the key clinical staff in the ED. The acronym of the project conceptualised the core messaging of the value of the patient to others and the vision of inclusion. Rounding with staff identified what was going well, not well and if additional resources were required when working with NOK. Evidence based practice and an understanding of the key issues of ensuring safe passage of patients out from the ED was encouraged before detainment under the MHA could end. This included a structured future MH plan. The key message was to ensure NOK were involved in the discharge of the patient whenever possible if the person was under the MHA (see Table 2).

**Table 2.** Strategy to engage next of kin

• Identification of patient preference of next of kin
• Phone contact with next of kin
• In department meeting when possible (next of kin may be present)
• Collaborative care planning, acknowledgement of current health needs, existing health care in the community and any concerns
• Warm handover on discharge
• Discharge into the face to face care of next of kin whenever possible

The vision was to increase the NOK participation rate for MHA patients who subsequently left the ED in the 3 months from July 2021. The patients presenting voluntarily were not a target group. However, clinicians were encouraged to use their discretion to engage next of kin in this group too.

A retrospective audit was undertaken to clarify if the 2021 intervention had been successful. All people seen under the MHA between the 3-month periods of July to September 2020 and July to September 2021 were compared. Chi-square (χ2) analysis was used to evaluate the change across the two periods. Admission rates of MH patients to Dubbo hospital for these periods were also evaluated to determine the potential impact of NOK engagement (as the impact of the WANTED project may generalise to all patients) with Chi-square (χ2) analysis. Qualitative rounding was undertaken with 12 key clinicians (across disciplines and departments). This focussed on identifying what had been successful in the project and what could be improved. Questions were also asked about the need for any additional resources.

## Results

File audit was conducted for the 3-month periods in 2020 and 2021 (Table 3) and cross checked by multiple reviewers to ensure data accuracy. The focused effort to increase NOK involvement for patients under the MHA rose from 63.2% to 90.8%. A chi-square test of independence was performed to examine the relationship between the year in which people were seen and if they had NOK involvement or not. The relationship between these variables was significant χ2 (1, *N*=166) =18.75, *p* = .000,015.

**Table 3.** Next of kin (NOK) Consulted for patients in the Emergency Department (ED) who presented under the mental health act (MHA)

Year (Jul-Sep)	Next of kin consulted	%	Not consulted	%	Total
Patients under the MHA					
2020	43	63.2	25	36.8	68
2021	89	90.8	9	9.2	98
Total	132		34		166
					χ2, *p* = .000015*

*significant as *p* value is less than 0.05.

Overall MH presentations to the Dubbo ED are reported (Table 4) for comparison. A chi-square test of independence was performed to examine the relationship between the year and if patients were admitted to Dubbo Hospital or not. The relationship between these variables was significant with a fall in admissions from the ED during the intervention period in 2021. χ2 (1, *N*=652) =8.32, *p* = .0039.

**Table 4.** Total mental health patients admitted to Dubbo Hospital from the emergency department (ED)

Year (Jul-Sep)	Admitted	%	Not admitted	%	Total
Mental health presentations -emergency department activity					
2020	133	39	208	61	341
2021	88	28.3	223	71.7	311
Total	221		431		652
					χ2, *p* = .0039*

*significant as *p* value is less than 0.05.

Four key qualitative themes were noted on rounding with the clinicians engaged in this project in the ED. Engaging NOK should generally be attempted for all MH patients in the ED, not just patients under the MHA. Secondly, this was a clear gap that had been addressed by a framework via this project. The third key response was that there had been a change in culture, that this was the new norm. Finally that NOK were seen as allies. Importantly, there was no negative feedback.

## Discussion

Transformation efforts may fail because there is not a sense of urgency, a guiding coalition (the local MH and ED executive) and a vision that is then communicated with others (the clinicians), who can then act on that vision to institutionally incorporate.^8^ This was the goal and frame of this quality improvement activity.

Via the purpose of this project staff became increasingly uneasy if they were unable to make contact with next of kin. See the patient vignette (Table 5).

**Table 5.** A case for consideration

Patient vignette
Lesley is an 18 year old who was brought in by police under the mental health Act after a phone call from the Aunt. There were concerns noted on the mental health document about a recent social media post mentioning suicide. Lesley reports superficial forearm and thigh cutting with non-lethal intent, while intoxicated earlier in the evening. Lesley lives six km from town. It is 4a.m. and no relatives can be contacted successfully
How might this case be approached in your facility to ensure the patient’s safety?

Beliefs that it may be more effort to engage NOK were addressed amid the clinician group. Dissatisfaction of both patients and their NOK cluster around the main theme of interpersonal dissatisfaction, rather than disappointment in the competency of clinicians.^9^ This may be due to limited opportunities for meaningful engagement or poorly framed expectations. In contrast, working with patients, as well as their loved ones, is now considered an integral part of both high quality care and improved patient safety across the spectrum of medical care.^10^ Patient engagement is known to enhance care, service delivery and governance.^11^ The project results are consistent with an emerging literature of inclusion of patients, their families and a co-design approach in health care.

Most importantly, in this project the co-design of present and future care plans with the patient themselves was key. Of doing with and not doing to, or for.^12^ Clinicians communicated with the patient that a safe plan for discharge, into the care of someone they identified, who potentially loved them, was expected as best practice health care.

## Limitations

Feedback was not collated from the patient and next of kin group and this is a limitation, but also a future opportunity. The authors also noted the lower number of MH presentations under the MHA in 2020 versus 2021 and hypothesised major social and behavioural changes including lockdowns and outbreaks, amid SARS-CoV-2.^13^

## Conclusion

The concept of always attempting to include the NOK became normative and institutionalised amid the cohort of MH patients under the MHA in the ED, with the consent of the person and communicated as a best practice value of the organisation. Clinicians reported that this practice also generalised to other MH patients seen within the ED. The hospital admission rate was lowered and the community treatment rate was increased within the context of this new milieu.
